# Lactobacilli displacement and *Candida albicans* inhibition on initial adhesion assays: a probiotic analysis

**DOI:** 10.1186/s13104-022-06114-z

**Published:** 2022-07-07

**Authors:** Robert Josue Rodríguez-Arias, Bryan Omar Guachi-Álvarez, Dominique Esther Montalvo-Vivero, António Machado

**Affiliations:** Colegio de Ciencias Biológicas y Ambientales COCIBA, Instituto de Microbiología, Laboratorio de Bacteriología, Calle Diego de Robles y Pampite, Universidad San Francisco de Quito USFQ, Quito, Ecuador

**Keywords:** *Candida albicans*, Initial adhesion, *Lactobacillus gasseri*, *Lactobacillus plantarum*, Probiotics

## Abstract

**Objective:**

This study evaluates the probiotic activity of three vaginal *Lactobacillus gasseri* (H59.2, IMAUFB014, and JCM1131) and one non-vaginal *L. plantarum* ATCC14917 against three *Candida albicans* (ATCC10231, candidiasis, and healthy vaginal microbiota). Displacement of lactobacilli and adhesion inhibition of *C. albicans* were evaluated on an abiotic surface through adhesion assays with different experimental settings (ES) through low (1.0E + 03 CFU/ml) and high (1.00E + 09 CFU/ml) levels of colonization. ES simulated dysbiosis (ES1 and ES4), candidiasis (ES2), and healthy vaginal microbiota (ES3).

**Results:**

At ES2 and ES3, *L. gasseri* H59.2 showed discrepant inhibition values among *C. albicans* isolates (ES2: *P* = 0.008, ES3: *P* = 0.030; two‐way ANOVA). *L. plantarum* was only displaced by 23%, 31%, 54%, and 94% against low and high levels of *C. albicans* ATCC10231. *L. plantarum* was less displaced, when compared to *L. gasseri* strains (ES1: 61–84%, ES2: 82–96%, ES3: 83–95%, and ES4: 73–97%), showing multiple statistical differences (ES1: *P* =  < 0.001, ES2: *P* = 0.003, and ES3: *P* =  < 0.001; two‐way ANOVA). *L. plantarum* also showed a superior inhibition of *C. albicans* ATCC10231 in ES1 (81%) and ES2 (58%) when compared to *L. gasseri* strains (ES1: 27–73%, *P* < 0.001; and ES2:1–49%, *P* < 0.001; two‐way ANOVA).

**Supplementary Information:**

The online version contains supplementary material available at 10.1186/s13104-022-06114-z.

## Introduction

The vaginal microbiota is colonized by several microorganisms [1], where commensal *Lactobacillus* species act as defense mechanism [2]. Lactobacilli can adhere and biosynthesize antimicrobial compounds reducing colonization by pathogens [1] associated with bacterial vaginosis, aerobic vaginitis, and candidiasis [3]. Thus, probiotics could be an efficient alternative to antimicrobial treatment, which reduces commensal microbiota and increases resistance [4, 5]. Lactobacilli may restore healthy microbiota, as postulated by Mitrea and colleagues [5]. Although *Candida* spp. is commensal, this genus can become an opportunistic pathogen in high levels [6, 7] leading to vulvovaginal candidiasis and evolving in more serious urinary tract infections and venereal diseases [8, 9]. Studies reported the application of lactobacilli biofilms and biosurfactants against pathogens [10–13]. However, these approaches are designed to treat established infections and do not endure in vaginal microbiota [14]. Another approach could be the colonization of the mucosal epithelia by new and more probiotic lactobacilli [15], allowing a permanent integration in the microbiota. However, the inhibition of the initial adhesion of pathogens by lactobacilli is not fully understood [16, 17]. It is important to compare the variability of vaginal and non-vaginal lactobacilli to inhibit the adhesion of pathogens and to avoid their displacement. This study evaluated the probiotic ability of three vaginal *L. gasseri* and one non-vaginal *L. plantarum* to protect an abiotic surface in initial adhesion assays, assessing the lactobacilli displacement and the inhibition of three *C. albicans* through different scenarios of dysbiosis conditions, candidiasis, and healthy vaginal microbiota.

## Main text

### Methods

From previous studies [3, 18], three *Lactobacillus gasseri* (H59.2, IMAUFB014, and JCM1131), two *Candida albicans* (one isolate from a healthy vaginal microbiota, and another from candidiasis), and *C. albicans* ATCC10231 and *L. plantarum* ATCC14917 were used in this study. *Lactobacillus* strains were grown in Man, Rogosa and Sharpe agar for 48 h at 37 °C under microaerophilic conditions [19, 20]. *C. albicans* strains were grown in Sabouraud Dextrose Agar at 37 °C for 18 h [19–21]. Brain Heart Infusion (BHI) broth was used for initial adhesion assays [20].

#### Initial adhesion assays

Each microorganism was concentrated in 5 ml of sterile phosphate-buffered saline (PBS) solution. Both suspensions were collected by centrifugation (4000 g, 12 min, at room temperature), and washed twice with PBS. The pellet was resuspended according to the growth curves to 1.0E + 03 colony-forming unit (CFU)/ml and 1.00E + 09 CFU/ml (Additional files 1 and 2) by optical density at 600 nm (OD600). Four experimental settings (ES) were made from concentration combinations (Additional file 3), varying on low levels of lactobacilli (1.00E + 03 CFU/ml) against low and high levels of *C. albicans* (ES1 and ES2, respectively) and then on high levels of lactobacilli (1.00E + 09 CFU/ml) against low and high levels of *C. albicans* (ES3 and ES4, respectively). These ES mimicked dysbiosis conditions (ES1 and ES4), candidiasis (ES2), and healthy vaginal microbiota (ES3). Initial adhesion assays were realized using a preincubation of lactobacilli for 4 h at 37 °C with 120 rpm [22, 23] and then evaluating the initial adhesion of *Candida albicans* with the pre-adhered lactobacilli during 30 min at same conditions [23–26], as illustrated in the flowchart (Additional file 4) [27, 28]. Non-adherent microorganisms were removed by PBS washing. All experimental assays were repeated three times on different days.

#### Microscopy analysis and cell quantification

After adhesion assay, a PBS washing step was carried out on coverslips, which were fixed with ethanol (96%; v/v) and stained with crystal violet at 3% for 1 min [29]. From each coverslip, 15 random fields were photographed in Olympus BX50 microscope under 1000x [23, 30] using the AmScope MU633-FL camera. The number of *Lactobacillus* spp. and *C. albicans* were counted from each picture (Additional file 5), being expressed as the number of cells per glass surface ± standard deviation (Additional file 6 and Additional file 7).

### Statistical analysis

The statistical analysis was realized through two-tailed ANOVA (ANalysis Of VAriance) with post-hoc Tukey HSD (Honestly Significant Difference) and Student *t*-test using JASP software version 0.13. ANOVA analysis evaluated differences in and between ES, post-hoc Tukey HSD test analyzed differences between species on the same ES, and Student *t*-test assessed differences between samples and controls. *P* values ≤ 0.050 were statistically significant.

### Results

**Fig. 1:**
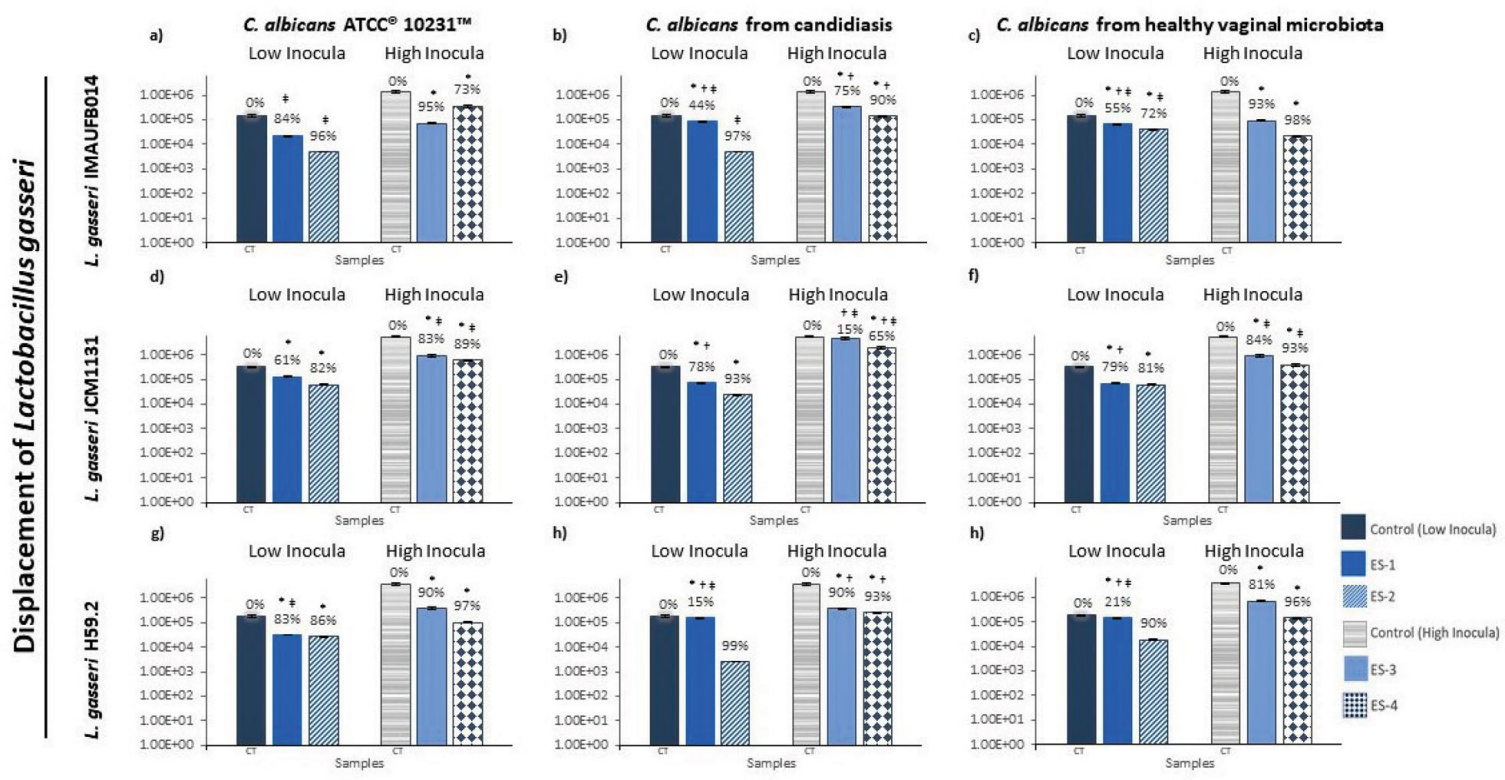
Displacement of *Lactobacillus gasseri* by *Candida albicans* obtained through initial adhesion assays. Displacement of *L. gasseri* by *C. albicans* after initial adhesion treatments with the experimental setting of high and low inoculum in the glass surface. The percentage of adhesion of *L. gasseri* is the result of the variation in the adhesion of *L. gasseri* and *C. albicans* strains to coverslip in comparison to controls (CT, 100% of adhesion) when incubated alone at the same conditions. Statistical analysis: *P < 0.05 when using t-student statistical analysis (95% confidence interval) for comparison of lactobacilli control and sample tested in the adhesion assay; ^†^*P* < 0.05 analyzed using two-tailed ANOVA statistical test (95% confidence interval) for comparison of displacement values from all lactobacilli strains induced by a certain *C. albicans* isolate tested in the adhesion assay; ^‡^*P* < 0.05 analyzed using two-tailed ANOVA statistical test (95% confidence interval) for comparison of displacement values from a certain strain of lactobacilli among all *C. albicans* isolates tested in the adhesion assay

Initial adhesion assays were realized through different experimental settings, simulating dysbiosis conditions (ES1 and ES4), candidiasis (ES2), and healthy vaginal microbiota (ES3). The lactobacilli displacement was evaluated on low levels (ES1 and ES2) against low and high concentrations of *C. albicans* and then on high levels (ES3 and ES4), as shown in Additional file 6. On low levels of lactobacilli, the displacement was between 15 and 99% (see Fig. 1). At ES1, *C. albicans* ATCC10231 induced bigger displacement of *L. gasseri* IMAUFB014 (84%; *P* = 0.010, two‐way ANOVA) and H59.2 (83%; *P* < 0.001, two‐way ANOVA) showing significant differences among *C. albicans* (Tukey's post hoc, *P* < 0.05). Likewise, all *C. albicans* showed to be statistically different in their displacement ability among the *L. gasseri*. *C. albicans* from healthy vaginal microbiota was able to displace 99% of *L. gasseri* IMAUFB014, while *C. albicans* isolated from candidiasis demonstrated 99% of displacement against *L. gasseri* JCM1131. At ES2, no significant differences were found in the displacement among *L. gasseri*. At ES3, *C. albicans* isolated from candidiasis showed statistical differences, evidencing a greater ability to displace *L. gasseri* H59.2 (90%; *P* < 0.001, two‐way ANOVA). *L. gasseri* JCM1131 showed only 15% of displacement by *C. albicans* isolated from candidiasis, being statistically different when compared to *C. albicans* ATCC10231 (83%; *P* = 0.001, Tukey's post hoc) and *C. albicans* isolated from healthy vaginal microbiota (84%; *P* < 0.001, Tukey's post hoc). At ES4, *L. gasseri* JCM1131 showed 65% of displacement by *C. albicans* isolated from candidiasis, but it only evidenced a significant difference against *C. albicans* isolated from healthy vaginal microbiota (93%; *P* = 0.045, Tukey's post hoc).

**Fig. 2:**
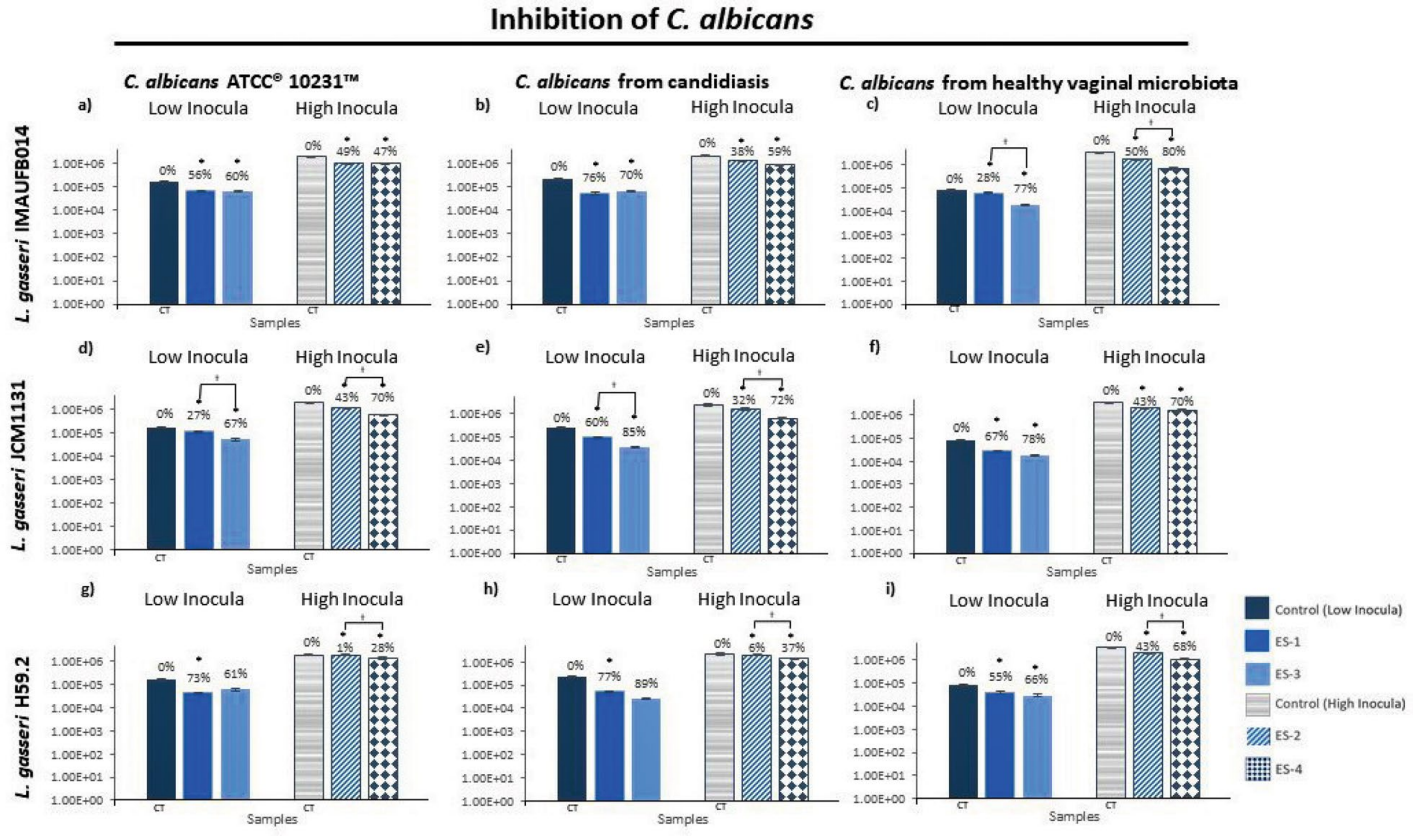
The probiotic activity of *Lactobacillus gasseri* against *Candida albicans* showed in initial adhesion assays. Inhibition of *C. albicans* by *L. gasseri* after initial adhesion treatments with the experimental setting of high and low inoculum in the glass surface. The percentage of adhesion of *C. albicans* is the result of the variation in the adhesion of *L. gasseri* and *C. albicans* strains to coverslip in comparison to controls (CT, 100% of adhesion) when incubated alone at the same conditions. Statistical analysis: *P < 0.05 when using t-student statistical analysis (95% confidence interval) for comparison of candida control and sample tested in the adhesion assay; ^†^*P* < 0.05 analyzed using two-tailed ANOVA statistical test (95% confidence interval) for comparison of inhibition values between experimental setting (ES) for each evaluated *C. albicans* isolated in the adhesion assay

The adhesion inhibition of *C. albicans* by *L. gasseri* was also evaluated (see Fig. 2). At ES1, *L. gasseri* JCM1131 and *L. gasseri* IMAUFB014 showed statistical differences among *C. albicans* (*L. gasseri* JCM1131 *P* = 0.006, and *L. gasseri* IMAUFB014 *P* = 0.002; two‐way ANOVA). *L. gasseri* JCM1131 evidenced the lowest inhibition rate against *C. albicans* ATCC10231 (27%), illustrating significant values when compared against *C. albicans* isolated from candidiasis (60%; *P* = 0.016, Tukey's post hoc) and *C. albicans* isolated from healthy vaginal microbiota (67%; *P* = 0.006, Tukey's post hoc). While *L. gasseri* IMAUFB014 showed a more efficient inhibition rate against *C. albicans* isolated from candidiasis (76%; *P* = 0.002, Tukey's post hoc). At ES2, *L. gasseri* H59.2 was the only strain to show statistically inhibition values among *C. albicans* isolates (*P* = 0.008; two‐way ANOVA). Again, at ES3, only *L. gasseri* H59.2 demonstrated a statistical difference in its inhibition ability (*P* = 0.030; two‐way ANOVA) against *C. albicans* ATCC10231 (61%) and *C. albicans* isolated from candidiasis (89%; *P* = 0.034, Tukey's post hoc). At ES4, all *C. albicans* showed significant inhibition rates (*C. albicans* ATCC10231: *P* = 0.010; *C. albicans* isolated from candidiasis: *P* = 0.011; *C. albicans* isolated from healthy microbiota: *P* = 0.025, two‐way ANOVA analysis). *L. gasseri* IMAUFB014 showed the highest inhibition rate against *C. albicans* isolated from healthy microbiota (80%), being statistically different to *C. albicans* ATCC10231 (47%; *P* = 0.016, Tukey's post hoc).

**Fig. 3:**
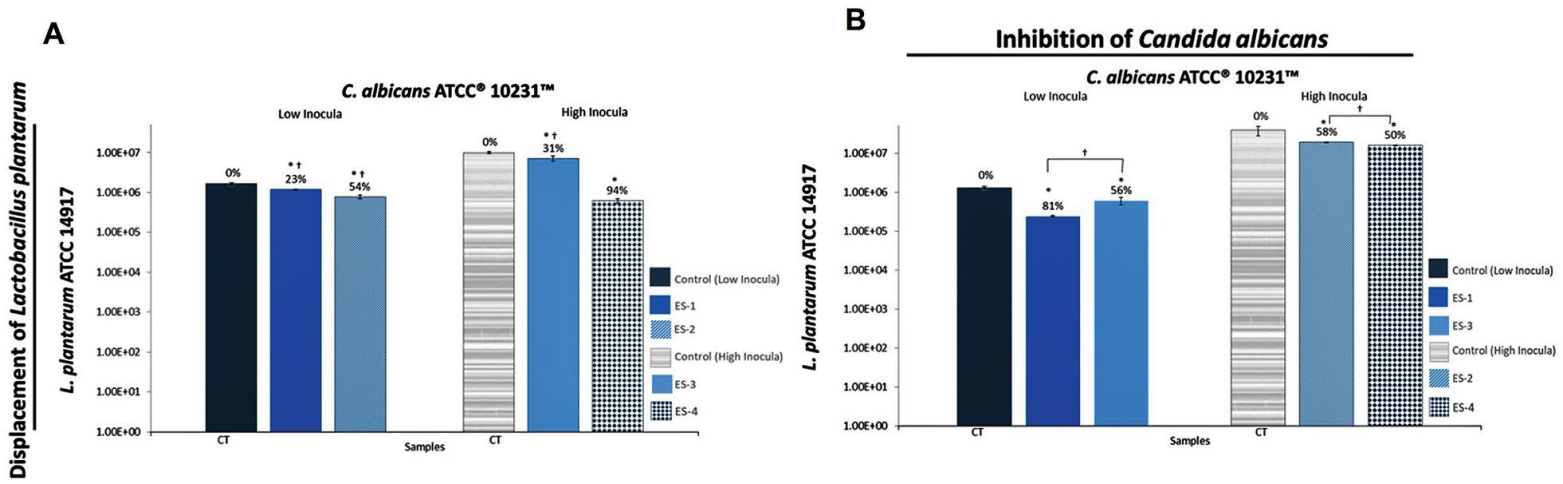
Preliminary analysis of the displacement of *Lactobacillus plantarum* by *Candida albicans* and its probiotic activity on *C. albicans* through initial adhesion assays. **A** Displacement of *L. plantarum* ATCC 14917 by *C. albicans* ATCC 10231 after initial adhesion treatments with the experimental setting of high and low inoculum in the glass surface. Statistical analysis: *P < 0.05 when using *t*-student statistical analysis (95% confidence interval) for comparison of lactobacilli control and sample tested in the adhesion assay; ^†^*P* < 0.05 analyzed using two-tailed ANOVA statistical test (95% confidence interval) for comparison of displacement values between *L. plantarum* and *L. gasseri* strains in the adhesion assay at same experimental setting. **B** Inhibition of *C. albicans* by *L. plantarum* after initial adhesion treatments with the experimental setting of high and low inoculum in the glass surface. Statistical analysis: *P < 0.05 when using t-student statistical analysis (95% confidence interval) for comparison of candida control and sample tested in the adhesion assay; ^†^*P* < 0.05 analyzed using two-tailed ANOVA statistical test (95% confidence interval) for comparison of inhibition values between experimental setting (ES) for each evaluated *C. albicans* ATCC 10231 isolated in the adhesion assay. No statistically significant values were found

Probiotic ability of *Lactobacillus plantarum* ATCC14917 was realized against *C. albicans* ATCC10231 and compared with *L. gasseri* evidencing significant displacement and inhibition values (see Additional file 7 and Fig. 3A). The displacement values of *L. plantarum* were 23% and 54% against low (ES1) and high (ES2) levels of *C. albicans*, respectively. These values were significantly inferior to *L. gasseri* (ES1: 61–99% and ES2: 82–96%), more exactly: *L. gasseri* IMAUFB014 (ES1: *P* < 0.001and ES2: *P* = 0.002, Tukey's post hoc); *L. gasseri* JCM1131 (ES1: *P* = 0.003 and ES2: *P* = 0.025, Tukey's post hoc); and *L. gasseri* H59.2 (ES1: *P* < 0.001 and ES2: *P* = 0.012, Tukey's post hoc). At ES3, *L. plantarum* was only displaced by 31% evidencing again a better resistance when compared to *L. gasseri* (ES3: 83–95%), specifically: *L. gasseri* IMAUFB014 (*P* < 0.001, Tukey's post hoc); *L. gasseri* H59.2 (*P* < 0.001, Tukey's post hoc); and *L. gasseri* JCM1131 (*P* = 0.001, Tukey's post hoc). At ES4, *L. plantarum* was displaced 94% without statistical differences. As shown in Fig. 3B, the adhesion inhibition of *C. albicans* by *L. plantarum* demonstrated a superior activity in ES1 (81%) and ES2 (58%) when compared to *L. gasseri* (ES1:27–73% and ES2:1–49%; both *P* < 0.001, two‐way ANOVA). At ES3 and ES4, *L. plantarum* showed the lowest inhibition rate (50–56%) without statistical differences.

### Discussion

The use of *Lactobacillus* is a low-risk alternative for antimicrobial resistance and its adverse effects [31]. It is well-known that a prerequisite for *C. albicans'* pathogenicity is the initial adhesion to host cells [32] before leading to genital or urinary tract infections [8, 9]. This study focused on the intrinsic probiotic activity of lactobacilli against *C. albicans*. Although studies reported lactobacilli biofilm/biosurfactant activities against pathogens [10–13], few authors evaluated the inhibition of the initial adhesion of pathogens [16, 17, 23, 33]. Some studies previously characterized the inhibition of the initial adhesion of certain pathogens, such as *Gardnerella vaginalis*, *Prevotella bivia*, *Mobiluncus mulieris* [23], *Listeria monocytogenes* [34], and *Streptococcus mutans* [17]. Recently, He et al. [35] evaluated the probiotic activities of *Lactobacillus* species on the inhibition of the initial adhesion of several pathogens. However, only *L. gasseri* demonstrated a more probiotic activity against *C. albicans*, when compared to *L. crispatus*. Our results agreed with He et al. [35], reporting differences in probiotic activity among *Lactobacillus* species/strains against *C. albicans* isolates. However, there is still scarce information about how these *Candida albicans* can be inhibited by *Lactobacillus* strains/species or even to displace different lactobacilli.

An alternative approach could be used by the colonization of new and more probiotic lactobacilli in this environment [15], being permanently assimilate in the vaginal microbiota. Once incorporated, certain lactobacilli species should be able to produce supernatant and eventually evolve in biofilm formation [15, 36], such as *L. plantarum*. So, the initial adhesion is a vital step for the human epithelial colonization and the inhibition of pathogens being worthy to study.

The present study evaluated three *L. gasseri* as inherent vaginal lactobacilli and a single *L. plantarum* (ATCC 14917) as a strong probiotic species atypical of the vaginal microbiota. Our results evidenced statistical differences between the displacement values of *L. gasseri* strains by the same *C. albicans* isolate and the variability of each *Lactobacillus* to inhibit different *C. albicans*. This variability agrees with a study realized by De Gregorio et al. [11] with *Lactobacillus crispatus* on the adhesion of *Candida* species, which evidenced the strain-specific probiotic activity of *L. crispatus*. So, it is plausible to assume that the remaining *Lactobacillus* species could also evidence discrepancies in their probiotic ability and displacement resistance against different *C. albicans* isolates, as proposed by Zangl et al. [15]. The application of different lactobacilli from other biological sources in the human epithelial colonization could increment the probiotic activity of the remaining commensal microbiota, as suggested in other studies [37–39]. These studies together with our results of low displacement values in *L. plantarum* against low or normal levels of *C. albicans* suggested the potential application of non-human lactobacilli to sustain a more resilient healthy microbiota. *L. plantarum* ATCC 14917 demonstrated high inhibition percentages of *C. albicans* ATCC 10231, being more efficient and statistically different when compared to *L. gasseri*. Our results surpassed the rates of inhibition on *C. albicans* reported by Dos Santos et al. [12]. Further studies should evaluate longitudinal colonization between non-vaginal lactobacilli and vaginal lactobacilli against *Candida* species in vitro assays.

## Limitations

There are some major limitations in this study: (1) it is a preliminary study realized on an abiotic surface and unable to establish an efficient report on human epithelial colonization; (2) the study did not evaluate the longitudinal colonization between lactobacilli and *C. albicans*; and (3) the study only compared the probiotic activity of *L. plantarum* against a single *Candida albicans*.

## Supplementary Information

**Additional file 1: Figure S1**. Comparison of growth calibration curves between *Lactobacillus gasseri* strains and *Lactobacillus plantarum* ATCC 14917 used in this study.

**Additional file 2: Figure S2**. Comparison of growth calibration curves between *C. albicans* strains used in this study.

**Additional file 3: Figure S3**. Representation of the experimental settings in adhesion assays simulating dysbiosis conditions (ES1 and ES4), candidiasis (ES2), and healthy vaginal microbiota (ES3).

**Additional file 4: Figure S4**. Illustration of the flowchart on the procedures used in the initial adhesion assays of the present study. The flowchart was realized using the online software CmapTools (https://cmap.ihmc.us/) [28].

**Additional file 5: Figure S5**. Comparison of sample for *L. gasseri* IMAUFB014 against *C. albicans* ATCC 10231 observed in the OLYMPUS BX50 microscope for each experimental setting (ES), evaluating the initial adhesion of *C. albicans* and the displacement of pre-adhered lactobacilli during 30 min. **A** Random field (1000x) of *L. gasseri* IMAUFB014 (1.00E + 03 CFU/ml) against *C. albicans* ATCC 10231 (1.00E + 03 CFU/ml) at ES1. **B** Random field (1000x) of *L. gasseri* IMAUFB014 (1.00E + 03 CFU/ml) against *C. albicans* ATCC 10231 (1.00E + 09 CFU/ml) at ES2. **C** Random field (1000x) of *L. gasseri* IMAUFB014 (1.00E + 09 CFU/ml) against *C. albicans* ATCC 10231 (1.00E + 03 CFU/ml) at ES3. **D** Random field (1000x) of *L. gasseri* IMAUFB014 (1.00E + 09 CFU/ml) against *C. albicans* ATCC 10231 (1.00E + 09 CFU/ml) at ES4.

**Additional file 6: Table S1.** Displacement of *Lactobacillus gasseri* by *Candida albicans* obtained through initial adhesion assays.

**Additional file 7: Table S2.** Displacement of *Lactobacillus plantarum* by *Candida albicans* obtained through initial adhesion assays.

## Data Availability

All the data supporting the study findings are within the manuscript. Additional detailed information and raw data are available from the corresponding author on reasonable request.

## Abbreviations

MRS: De Man, Rogosa and Sharpe agar
SDA: Sabouraud dextrose agar
BHI: Brain heart infusion
PBS: Phosphate buffered saline
CFU: Colony-forming unit
ES: Experimental setting
ANOVA: Analysis of variance
Tukey HSD: Tukey honestly significant difference test
