# Supplementary figures and images for: Lactobacilli displacement and Candida albicans inhibition on initial adhesion assays: a probiotic analysis
Source: BMC Res Notes. 2022 Jul 7;15:239. doi: 10.1186/s13104-022-06114-z (PMC9264498; doi:10.1186/s13104-022-06114-z)

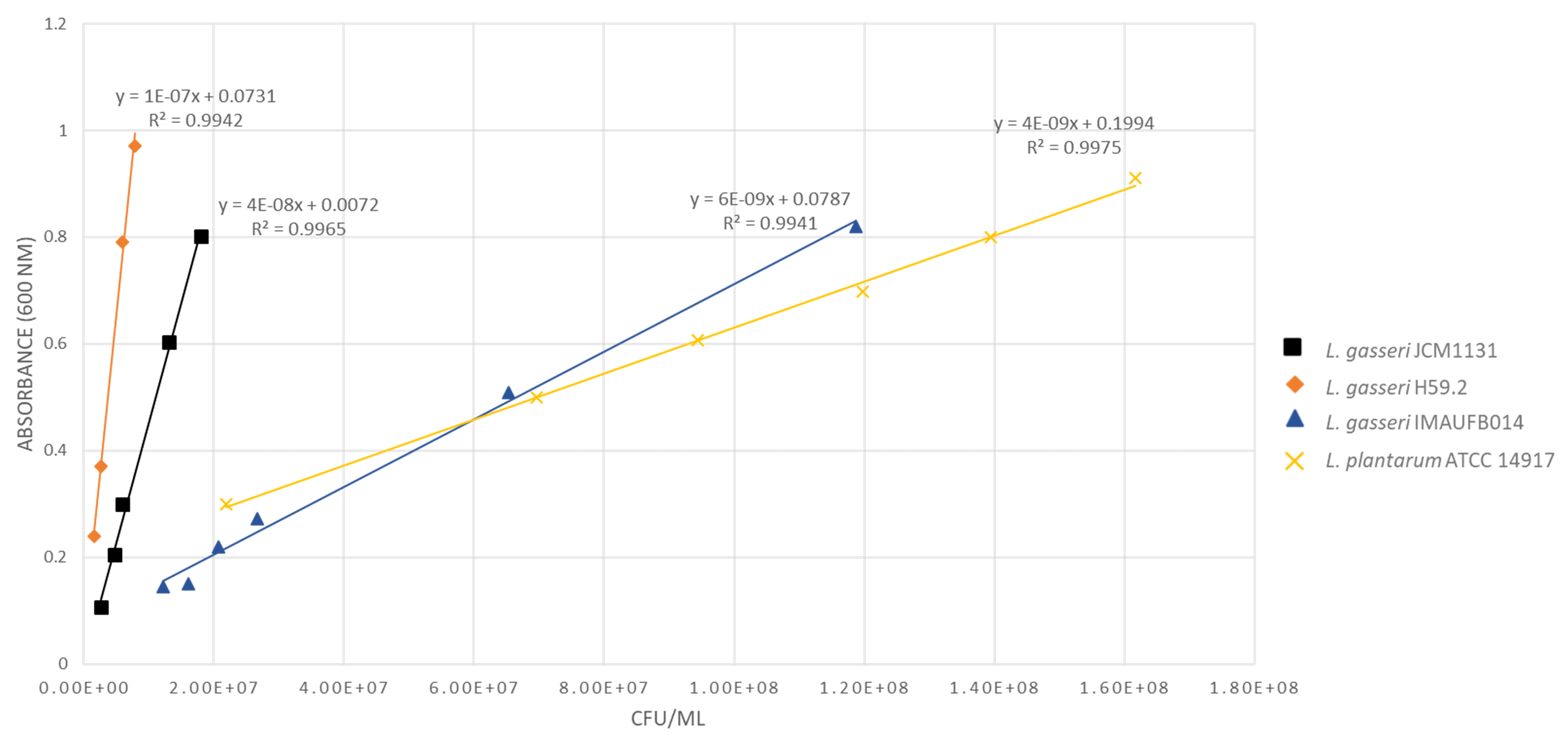

Supplement: Supplementary file 1 — Additional file 1: Figure S1. Comparison of growth calibration curves between Lactobacillus gasseri strains and Lactobacillus plantarum ATCC 14917 used in this study. [file 13104_2022_6114_MOESM1_ESM.png]

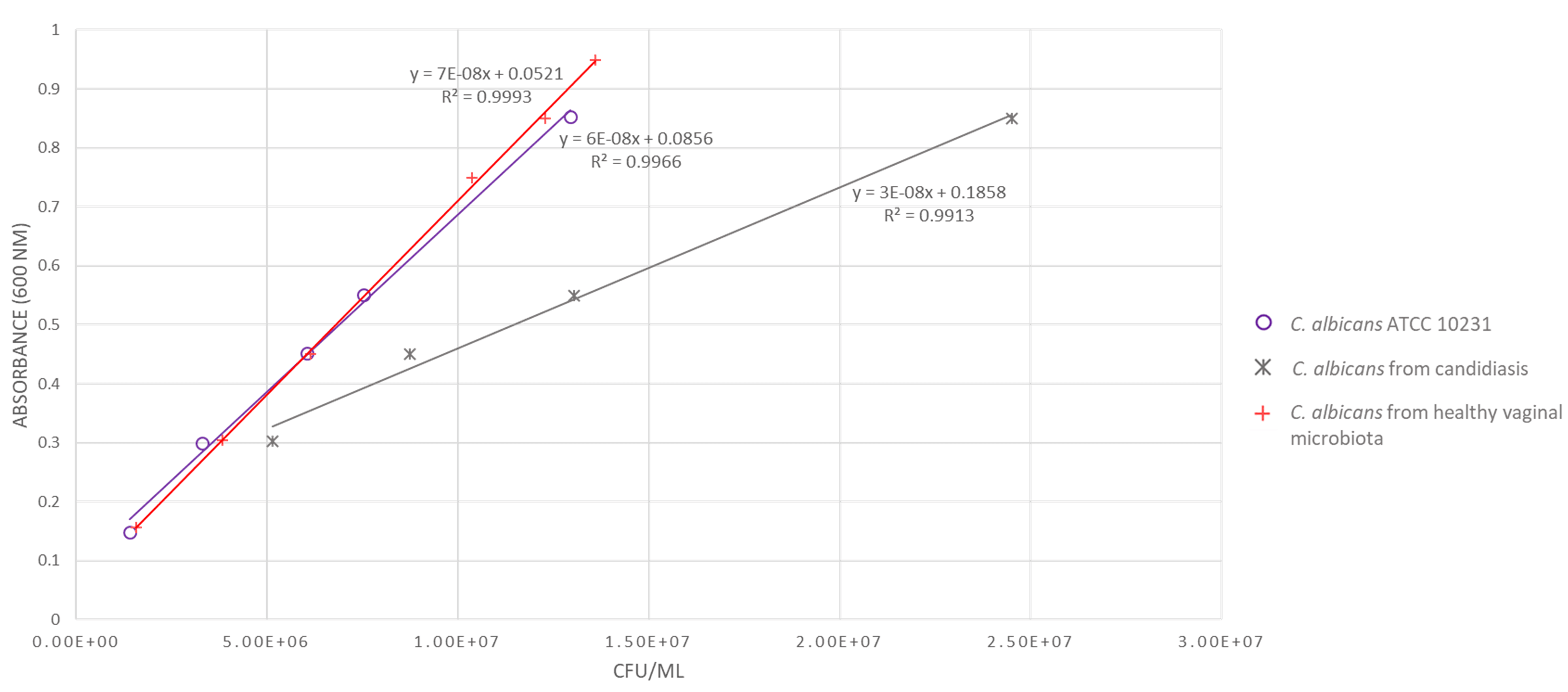

Supplement: Supplementary file 2 — Additional file 2: Figure S2. Comparison of growth calibration curves between C. albicans strains used in this study. [file 13104_2022_6114_MOESM2_ESM.png]

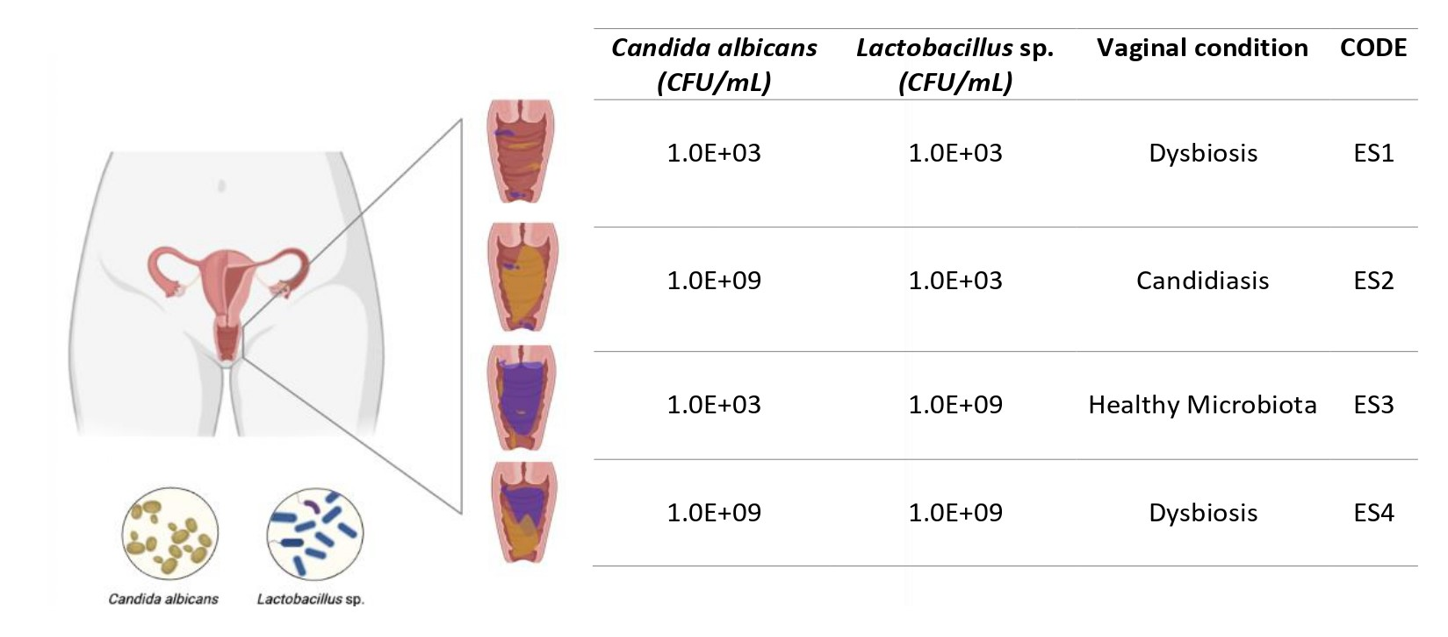

Supplement: Supplementary file 3 — Additional file 3: Figure S3. Representation of the experimental settings in adhesion assays simulating dysbiosis conditions (ES1 and ES4), candidiasis (ES2), and healthy vaginal microbiota (ES3). [file 13104_2022_6114_MOESM3_ESM.png]

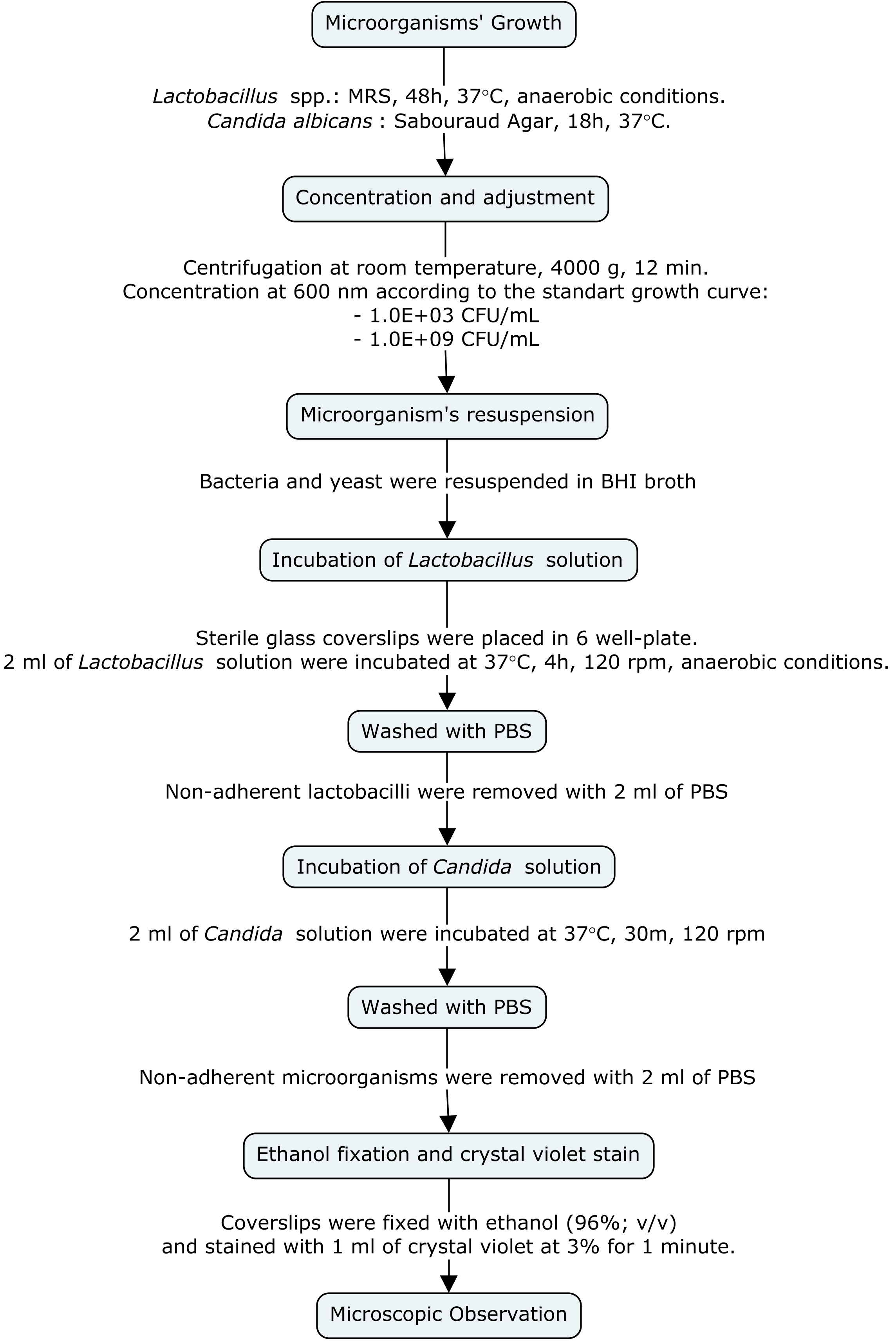

Supplement: Supplementary file 4 — Additional file 4: Figure S4. Illustration of the flowchart on the procedures used in the initial adhesion assays of the present study. The flowchart was realized using the online software CmapTools (https://cmap.ihmc.us/) [28]. [file 13104_2022_6114_MOESM4_ESM.png]

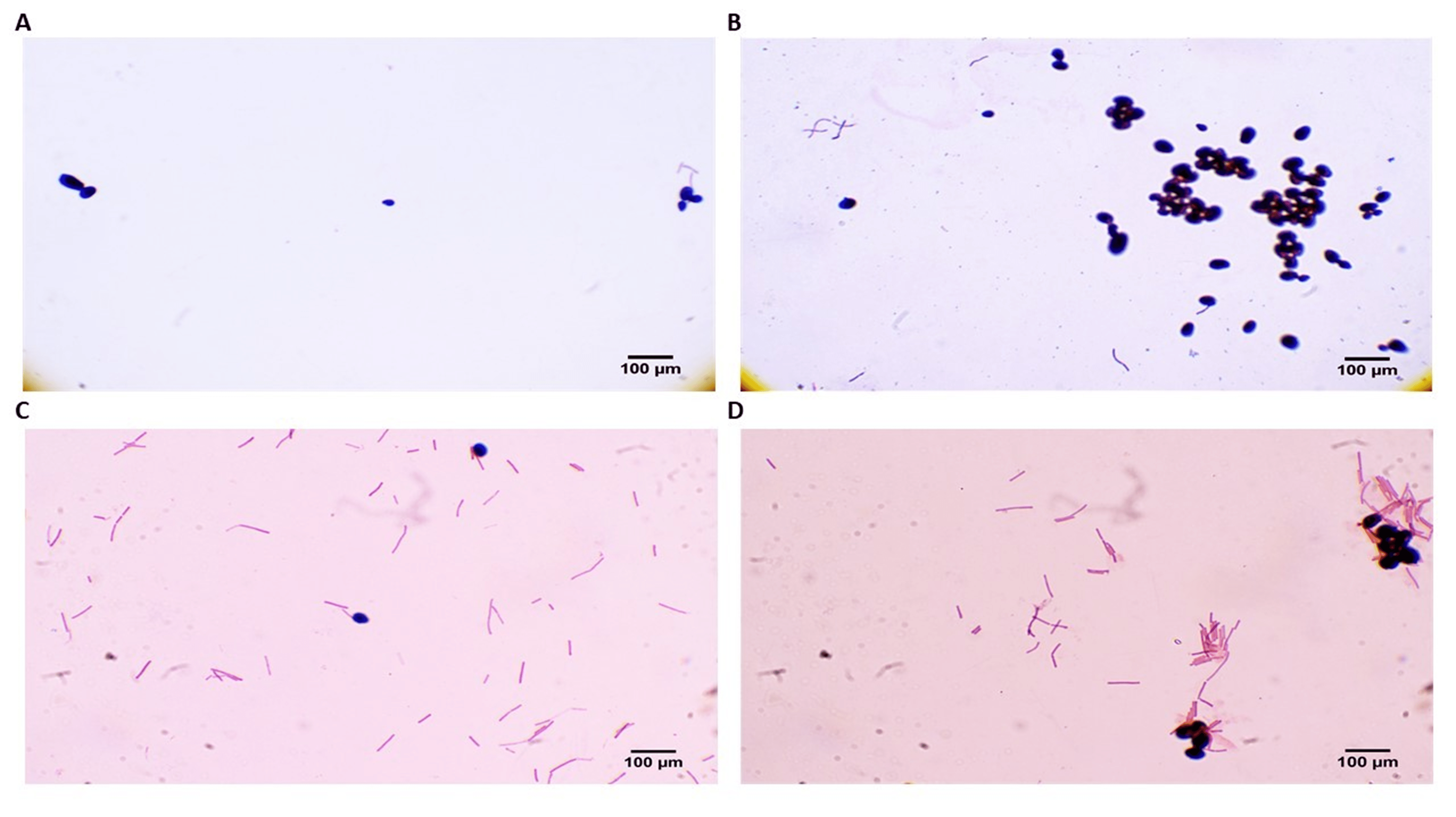

Supplement: Supplementary file 5 — Additional file 5: Figure S5. Comparison of sample for L. gasseri IMAUFB014 against C. albicans ATCC 10231 observed in the OLYMPUS BX50 microscope for each experimental setting (ES), evaluating the initial adhesion of C. albicans and the displacement of pre-adhered lactobacilli during 30 min. A Random field (1000x) of L. gasseri IMAUFB014 (1.00E + 03 CFU/ml) against C. albicans ATCC 10231 (1.00E + 03 CFU/ml) at ES1. B Random field (1000x) of L. gasseri IMAUFB014 (1.00E + 03 CFU/ml) against C. albicans ATCC 10231 (1.00E + 09 CFU/ml) at ES2. C Random field (1000x) of L. gasseri IMAUFB014 (1.00E + 09 CFU/ml) against C. albicans ATCC 10231 (1.00E + 03 CFU/ml) at ES3. D Random field (1000x) of L. gasseri IMAUFB014 (1.00E + 09 CFU/ml) against C. albicans ATCC 10231 (1.00E + 09 CFU/ml) at ES4. [file 13104_2022_6114_MOESM5_ESM.png]
