# Supplementary material for: Lactobacilli displacement and Candida albicans inhibition on initial adhesion assays: a probiotic analysis
Source: BMC Res Notes. 2022 Jul 7;15:239. doi: 10.1186/s13104-022-06114-z (PMC9264498; doi:10.1186/s13104-022-06114-z)
Supplement: Supplementary file 6 — Additional file 6: Table S1. Displacement of Lactobacillus gasseri by Candida albicans obtained through initial adhesion assays. [file 13104_2022_6114_MOESM6_ESM.docx]

**Supplementary Table 1.** Displacement of *Lactobacillus gasseri* by *Candida albicans* obtained through initial adhesion assays.

|  | | **Experimental setting (ES)** | | | | | | | |
| --- | --- | --- | --- | --- | --- | --- | --- | --- | --- |
| **Microorganisms** | | **1** | | **2** | | **3** | | **4** | |
|  |  | SAMPLE  (N. of cells per glass surface) | DISPL. (%) | SAMPLE  (N. of cells per glass surface) | DISPL. (%) | SAMPLE  (N. of cells per glass surface) | DISPL. (%) | SAMPLE  (N. of cells per glass surface) | DISPL. (%) |
| *L. gasseri* IMAUFB014 | *C. albicans* ATCC® 10231™ | 2.25E+04 (±2.66E+00) ^b^ | 84  (±0.08) | 5.01E+03  (±8.85E-11) ^b^ | 96  (±0.08) | 7.27E+04 (±4.34E+03) ^a^ | 95  (±0.09) | 3.58E+05 (±3.19E+04) ^a^ | 73  (±0.09) |
|  | *C. albicans* from candidiasis | 8.35E+04 (±8.05E+03) ^a,b,c^ | 44  (±0.10) | 5.01E+03  (±7.23E-11) ^b^ | 97  (±0.08) | 3.43E+05 (±5.01E+03) ^a,c^ | 75  (±0.08) | 1.39E+05 (±1.77E+03) ^a,c^ | 90  (±0.07) |
|  | *C. albicans* from healthy vaginal microbiota | 6.60E+00 (±1.45E+03) ^a,b,c^ | 99  (±0.08) | 4.09E+04 (±1.45E+03) ^a,b^ | 72  (±0.08) | 9.10E+04 (±7.65E+03) ^a^ | 93  (±0.09) | 2.13E+04 (±1.77E+03) ^a^ | 98  (±0.09) |
| *L. gasseri* JCM1131 | *C. albicans* ATCC® 10231™ | 1.27E+05 (±1.13E+04) ^a^ | 61  (±0.07) | 6.01E+04 (±3.54E+03) ^a^ | 82  (±0.06) | 9.33E+05 (±8.67E+04) ^a,b^ | 83  (±0.08) | 5.90E+05 (±5.49E+04) ^a,b^ | 89  (±0.08) |
|  | *C. albicans* from candidiasis | 7.27E+00 (±3.54E+03) ^a,c^ | 99  (±0.06) | 2.38E+04 (±1.77E+03) ^a^ | 93  (±0.06) | 4.69E+06 (±4.32E+05) ^b,c^ | 15  (±0.11) | 1.94E+06 (±1.57E+05) ^a,b,c^ | 65  (±0.09) |
|  | *C. albicans* from healthy vaginal microbiota | 6.93E+04 (±5.21E+03) ^a,c^ | 79  (±0.06) | 6.14E+04 (±1.77E+03) ^a^ | 81  (±0.06) | 8.96E+05 (±7.27E+04) ^a,b^ | 84  (±0.08) | 3.82E+05 (±3.98E+04) ^a,b^ | 93  (±0.08) |
| *L. gasseri* H59.2 | *C. albicans* ATCC® 10231™ | 3.01E+04  (±5.11E-12) ^a,b^ | 83  (±0.09) | 2.63E+04 (±1.77E+03) ^a^ | 86  (±0.09) | 3.73E+05 (±3.90E+04) ^a^ | 90  (±0.08) | 1.00E+05 (±4.34E+03) ^a^ | 97  (±0.08) |
|  | *C. albicans* from candidiasis | 1.54E+05 (±1.77E+03) ^a,b,c^ | 15  (±0.09) | 2.51E+03 (±0.00E+00) | 99  (±0.09) | 3.73E+05 (±2.71E+04) ^a,c^ | 90  (±0.08) | 2.58E+05 (±2.13E+04) ^a,c^ | 93  (±0.08) |
|  | *C. albicans* from healthy vaginal microbiota | 1.44E+05  (±1.16E+04) ^a,b,c^ | 21  (±0.10) | 1.84E+04 (±1.45E+03) | 90  (±0.09) | 6.91E+05 (±3.65E+04) ^a^ | 81  (±0.08) | 1.44E+05 (±1.77E+03) ^a^ | 96  (±0.08) |

**Sample**: The amount of *L. gasseri* adhered to the abiotic glass surface after initial adhesion assays of *L. gasseri* vs *C. albicans*.

**DISPL %:** percentage of *L. gasseri* displaced at the end of the initial adhesion assays.

**ES1**: *L. gasseri* (1.00E+03 CFU/ml) & *C. albicans* (1.00E+03 CFU/ml).

**ES2**: *L. gasseri* (1.00E+03 CFU/ml) & *C. albicans* (1.00E+09 CFU/ml).

**ES3**: *L. gasseri*. (1.00E+09 CFU/ml) & *C. albicans* (1.00E+03 CFU/ml).

**ES4**: *L. gasseri*. (1.00E+09 CFU/ml) & *C. albicans* (1.00E+09 CFU/ml).

The experimental positive controls (N. of cells per glass surface) for the high and low inoculums of ***L. gasseri*** obtained in this study were as follows: IMAUFB014 1.34 E + 06 (± 1.45 E + 05) & 1.48 E + 05 (± 1.48 E + 04 ); JCM1131 5.54 E + 06 (± 5.62 E + 05) & 3.27 E + 05 (± 2.53 E + 04); H59.2 3.70 E + 06 (± 3.51 E + 05) & 1.82 E + 05 (± 1.87 E + 04).

The experimental positive controls (N. of cells per glass surface) for the high and low inoculums of ***C. albicans*** obtained in this study were as follows: ATCC 10231 1.95 E + 06 (± 1.86 E + 05) & 1.60 E + 05 (± 1.49 E + 04); from candidiasis 2.16 E + 06 (± 1.84 E + 05) & 2.30 E + 05 (± 1.70 E + 04); from healthy vaginal microbiota 3.41 E + 06 (± 2.99 E + 05) & 8.52 E + 05 (± 7.60 E + 03).

All negative controls of ***L. gasseri*** and ***C. albicans*** showed no adhered cells on the abiotic surface, being considered as 0.00 E + 00 (± 0.00 E + 00) when compared to positive and samples in the initial adhesion assays.

**Statistical analysis:** ^a^ *P* < 0.05 when using *t*-student statistical analysis (95% confidence interval) for comparison of lactobacilli control and sample tested in the adhesion assay; ^b^ *P* < 0.05 analyzed using two-tailed ANOVA statistical test (95% confidence interval) for comparison of displacement values from a certain strain of lactobacilli among all *C. albicans* isolates tested in the adhesion assay; ^c^ *P* < 0.05 analyzed using two-tailed ANOVA statistical test (95% confidence interval) for comparison of displacement values from all lactobacilli strains induced by a certain *C. albicans* isolate tested in the adhesion assay.
