# Supplementary material for: Lactobacilli displacement and Candida albicans inhibition on initial adhesion assays: a probiotic analysis
Source: BMC Res Notes. 2022 Jul 7;15:239. doi: 10.1186/s13104-022-06114-z (PMC9264498; doi:10.1186/s13104-022-06114-z)
Supplement: Supplementary file 7 — Additional file 7: Table S2. Displacement of Lactobacillus plantarum by Candida albicans obtained through initial adhesion assays. [file 13104_2022_6114_MOESM7_ESM.docx]

**Supplementary Table 2.** Displacement of *Lactobacillus plantarum* by *Candida albicans* obtained through initial adhesion assays.

| **Microorganisms** | | **Experimental setting (ES)** | | | | | | | |  |
| --- | --- | --- | --- | --- | --- | --- | --- | --- | --- | --- |
|  |  | **1** | | **2** | | **3** | | **4** | |  |
|  |  |  |  |  |  |  |  |  |  |  |
|  |  | SAMPLE | DISPL. (%) | SAMPLE | DISPL. (%) | SAMPLE | DISPL. (%) | SAMPLE | DISPL. (%) |  |
|  |  | (N. of cells per glass surface) |  | (N. of cells per glass surface) |  | (N. of cells per glass surface) |  | (N. of cells per glass surface) |  |  |
| *L. plantarum ATCC 14917* | *C. albicans* ATCC® 10231™ | 1.20E+06 (1.27E+04) ^a,b^ | 23 (±0.82) | 7.78E+05 (9.23E+04) ^a,b^ | 54 (±5.44) | 7.05E+06 (1.01E+06) ^a,b^ | 31 (±9.82) | 6.16E+05 (7.74E+04) ^a^ | 94 (±0.81) |  |

**Sample**: the amount of *L. plantarum* adhered to the abiotic glass surface after initial adhesion assays of *L. plantarum* vs *C. albicans*.

**DISPL %:** percentage of *L. plantarum* displaced at the end of the initial adhesion assays.

**ES1**: *L. plantarum* (1.00E+03 CFU/ml) & *C. albicans* (1.00E+03 CFU/ml).

**ES2**: *L. plantarum* (1.00E+03 CFU/ml) & *C. albicans* (1.00E+09 CFU/ml).

**ES3**: *L. plantarum*. (1.00E+09 CFU/ml) & *C. albicans* (1.00E+03 CFU/ml).

**ES4**: *L. plantarum*. (1.00E+09 CFU/ml) & *C. albicans* (1.00E+09 CFU/ml).

The experimental positive controls (N. of cells per glass surface) for the high and low inoculums of *L. plantarum* obtained in this study were as follows: ATCC 14917 9.92E+06 (± 4.80E+05) & 1.63E+06 (± 9.85E+04).

The experimental positive controls (N. of cells per glass surface) for the high and low inoculums of *C. albicans* obtained in this study were as follows: ATCC 10231 3.87E+07 (± 1.04E+07) & 1.30E+06 (± 8.95E+04).

All negative controls of *L. plantarum* and *C. albicans* showed no adhered cells on the abiotic surface, being considered as 0.00 E + 00 (± 0.00 E + 00) when compared to positive and samples in the initial adhesion assays.

**Statistical analysis:** ^a^ *P* < 0.05 when using *t*-student statistical analysis (95% confidence interval) for comparison of lactobacilli control and sample tested in the adhesion assay; ^b^ *P* < 0.05 analyzed using two-tailed ANOVA statistical test (95% confidence interval) for comparison of displacement values between *L. plantarum* and *L. gasseri* strains in the adhesion assay at same experimental setting.
